# Effects of Omega-3 and Antioxidant Cocktail Supplement on Prolonged Bed Rest: Results from Serum Proteome and Sphingolipids Analysis

**DOI:** 10.3390/cells11132120

**Published:** 2022-07-05

**Authors:** Pietro Barbacini, Dieter Blottner, Daniele Capitanio, Gabor Trautmann, Katharina Block, Enrica Torretta, Manuela Moriggi, Michele Salanova, Cecilia Gelfi

**Affiliations:** 1 Department of Biomedical Sciences for Health, University of Milan, Via Luigi Mangiagalli 31, 20133 Milan, Italy; pietro.barbacini@unimi.it (P.B.); daniele.capitanio@unimi.it (D.C.); manuelamoriggi@yahoo.it (M.M.); 2 Institute of Integrative Neuroanatomy, Charité—Universitätsmedizin Berlin, Corporate Member of Freie Universität Berlin, Humboldt-Universität zu Berlin, and Berlin Institute of Health, 10115 Berlin, Germany; dieter.blottner@charite.de (D.B.); gabor.trautmann@charite.de (G.T.); michele.salanova@charite.de (M.S.); 3 Center of Space Medicine Berlin, 10115 Berlin, Germany; katharina.block@charite.de; 4 IRCCS Istituto Ortopedico Galeazzi, Via Riccardo Galeazzi 4, 20161 Milan, Italy; enrica.torretta@grupposandonato.it

**Keywords:** bed rest, *S*-nitrosylation, RONS, antioxidant, omega-3, serum apolipoproteins, iodoTMT, TMT, serum lipid profile, sphingolipids

## Abstract

Physical inactivity or prolonged bed rest (BR) induces muscle deconditioning in old and young subjects and can increase the cardiovascular disease risk (CVD) with dysregulation of the lipemic profile. Nutritional interventions, combining molecules such as polyphenols, vitamins and essential fatty acids, can influence some metabolic features associated with physical inactivity and decrease the reactive oxidative and nitrosative stress (RONS). The aim of this study was to detect circulating molecules correlated with BR in serum of healthy male subjects enrolled in a 60-day BR protocol to evaluate a nutritional intervention with an antioxidant cocktail as a disuse countermeasure (Toulouse COCKTAIL study). The serum proteome, sphingolipidome and nitrosoproteome were analyzed adopting different mass spectrometry-based approaches. Results in placebo-treated BR subjects indicated a marked decrease of proteins associated with high-density lipoproteins (HDL) involved in lipemic homeostasis not found in the cocktail-treated BR group. Moreover, long-chain ceramides decreased while sphingomyelin increased in the BR cocktail-treated group. In placebo, the ratio of *S*-nitrosylated/total protein increased for apolipoprotein D and several proteins were over-nitrosylated. In cocktail-treated BR subjects, the majority of protein showed a pattern of under-nitrosylation, except for ceruloplasmin and hemopexin, which were over-nitrosylated. Collectively, data indicate a positive effect of the cocktail in preserving lipemic and RONS homeostasis in extended disuse conditions.

## 1. Introduction

Physical inactivity or BR during hospitalization contributes to the functional decline of older patients [1], with increased CVD risk mainly associated with lower HDL levels [2]. The correlation between HDL cholesterol and physical activity has been investigated since the 1990s in young adults, where it was described for the first time as an HDL-cholesterol decrease and a dysregulation of the lipemic profile in healthy young subjects enrolled in a 20-day BR protocol, providing evidence that bedridden condition is causative of these changes [3]. From this observation we can presume that the loss of muscle mass and force, identified in several studies based on prolonged BR, is not the only detrimental effect of inactivity but also the cardiovascular system is involved [4,5]. It has been demonstrated that resistive countermeasures can prevent muscle remodeling but appear ineffective in reducing the CVD risk associated with decreased HDL levels [6]. It is now clear that BR studies of young healthy subjects can offer the opportunity to identify specific circulating markers associated with decreased HDL levels and are an appropriate model to investigate the effect of nutritional and resistive or vibration countermeasures to be transferred in prolonged spaceflight missions and clinical settings to prevent sarcopenia and reduce CVD risk.

Nutritional intervention based on the supplement of a single molecule (i.e., vitamin E, selenium or omega-3) provided significant results in animal models and in patients affected by muscle disorders or obesity [7,8,9]. However, they are poorly described in healthy young subjects. Vitamin E and selenium have been adopted as antioxidant scavengers to prevent muscle atrophy in aged subjects and dystrophic muscle [10,11], while omega-3 (ω-3) supplementation influences muscle metabolism and lipogenesis with positive results in young obese subjects [7]. Unfortunately, contradictory results were found concerning inflammation, in which a positive action of ω-3 was observed in muscle-related parameters in the elderly and in patients with chronic renal failure [12]. In healthy subjects, a high dose of ω-3 supplementation did not result in appreciable effects on muscle metabolism or muscle mass and function under basal conditions [13]. However, the treatment appears effective in decreasing atrophy, low-grade inflammation and in activating protein synthesis in the hind limb immobilized animal model [14]. In human subjects with hypertriglyceridemia, ω-3 supplement improved the plasma lipid profile (decrease in VLDL and APOB100) and ameliorated the sphingolipid content of VLDL, decreasing ceramide and increasing sphingomyelin levels [15], suggesting that this nutritional supplementation can have a positive effect in modulating the lipid homeostasis, although not clearly defined in healthy subjects.

What appears clear from previous studies on the use of specific molecules to counteract the detrimental effects of inactivity on muscle is that none of them alone can provide protection, suggesting that several mechanisms are involved, and each one of them has to be specifically targeted to reach a synergistic result leading to the maintenance of the muscle function. Recent observations from human and animal studies [16,17,18] demonstrated that the additive and synergistic effects of a combined use of molecules such as polyphenols, vitamins and essential fatty acids can influence some metabolic features associated with physical inactivity [19]. In muscle, previous studies from the Toulouse (Cocktail) BR study indicated that the combined use of ω-3, polyphenols and vitamins influences protein post-translational modifications targeting the redox homeostasis balance [20]. Results from nitrosoproteome, protein ubiquitination and protein carbonylation on protein extracts from *vastus lateralis* muscle biopsies indicated increased nitrosative redox homeostasis, lower protein ubiquitination and lower carbonylation, suggesting protection from oxidative damage in subjects under cocktail treatment. However, the preservation of lipemic homeostasis at the systemic level was not addressed [20,21].

The present study detects, in human blood serum, molecules able to monitor the evolution of lipemic changes possibly correlated with increased cardiovascular risk in healthy male subjects enrolled in the 60-day BR protocol with and without nutritional intervention by antioxidant cocktail treatment (Toulouse COCKTAIL study). To provide a comprehensive picture, three different high-resolution biochemical/omics tools were used for a global analysis of human blood serum samples (obtained by venipunctures) drawn from BR subjects shortly before BR start (PRE), at BR end (POST) and following 2 days of recovery (REC). We analyzed the serum proteome by Tandem mass Tag LC-MS/MS (TMT LC-MS/MS), the sphingolipid profile by LC-MS/MS, and the nitrosoproteome by Iodoacetyl-TMT LC-MS/MS (iodoTMT LC-MS/MS). Results indicated a marked decrease of proteins associated with HDL and involved in lipemic homeostasis in placebo-treated BR subjects that otherwise were not found in the cocktail-treated BR group. This study allowed to define a set of molecules that can be monitored for the effects of prolonged BR and profile the response, at least at systemic levels, to specific nutritional treatments and physical countermeasures under extended conditions of disuse and immobilization.

## 2. Materials and Methods

### 2.1. Subjects Recruitment and Study Design

Twenty males, non-smokers, active subjects without physical signs or medical history of neuromuscular disorders, were selected for the study. The BR part of the study took place at the Space Clinic of the Institute of Space Medicine and Physiology (Medes-IMPS, Rangueil Hospital) in Toulouse (France) and consisted of two different campaigns of 60 days, of 6° head-down tilt BR [21].

Anthropometric characteristics, including age (mean 34 ± 7.8 y.o.), height (mean 176 ± 5 cm), weight (mean 73.5 ± 6.1 kg), and body mass index (BMI) (mean 23.7 ± 1.5) were obtained from each participant [21]. Subjects were not under medications or were taking any drugs at the moment of the test. Written informed consent was obtained ahead of the study from each participant. The study was sponsored by the European Space Agency (ESA) and the French National Space Agency (CNES), approved by the Ethics Committee CPP Sud-Ouest et Outre-Mer I, France (number ID RCB: 2016-A00401–50), and all procedures were conducted following the guidelines of the Declaration of Helsinki [21].

Subjects were assigned randomly and on a double-blind basis, either to the placebo (*n* = 10) or the cocktail (*n* = 10) groups. A period of 14 ambulatory days before BR was used to collect baseline data followed by 60 days of 6° head-down tilt BR, a well-accepted spaceflight analogue to simulate microgravity-associated changes in the largely inactive healthy human body. BR subjects had their diet monitored at all times; meals were defined by the MEDES nutritionist and provided by the CHU Rangueil Hospital, Toulouse, France. During the 60 days BR, the Cocktail BR group received a daily oral dose of antioxidant/anti-inflammatory nutrient. The nutrient composition of the cocktail comprised polyphenols mix (741 mg), vitamin E (138 mg), selenium (80 µg) and ω-3 (2.1 g). After the end of BR, subjects remained in the facility for 14 days of reambulation and returned to the facility several times for follow-up monitoring [21]. Serum samples employed for the study were obtained 6 days before BR (PRE), at the 58th day of BR (POST) and 2 days after recovery/reambulation (REC).

### 2.2. Reagents and Chemicals

UPLC-MS Methanol, UPLC-MS grade water, triethylammonium bicarbonate (TEAB), tris(2 carboxyethyl)phosphine (TCEP), iodoacetamide, methyl methanethiosulfonate (MMTS) and trypsin were from Thermo Fisher Scientific (Waltham, MA, USA). Chloroform, 3,5-Di-tert-4-butylhydroxytoluene (BHT), LC-MS acetonitrile, LC-MS acetone and ammonium formate were from Sigma-Aldrich (Saint Louis, MO, USA). Potassium hydroxide (KOH), ammonium formate, sodium ascorbate, Tris-buffered saline (TBS), 4-(2-hydroxyethyl)-1-piperazineethanesulfonic acid (HEPES), Ethylenediaminetetraacetic acid (EDTA), neucuproine, sodium dodecyl sulfate (SDS), dithiothreitol (DTT) and ammonium bicarbonate (AMBIC) were from Merk Millipore (Burlington, MA, USA). Acetic and formic acid were from Fluka-Analytical (Honeywell, Morris Plains, NJ, USA).

### 2.3. Tandem Mass Tag (TMT) Sample Preparation

Proteins from serum samples were depleted from albumin with Pierce Albumin depletion Kit (Thermo Fisher Scientific), according to manufacturer instruction. Albumin-depleted proteins were acetone precipitated and resuspended in 100 mM TEAB. 100 µg (n. 5 Cocktail treated group and n. 5 Placebo treated group) of albumin-depleted proteins from both placebo and cocktail groups were adjusted to 100 μL with 100 mM TEAB, reduced with 200 mM TCEP, and alkylated with 375 mM iodoacetamide. Proteins were purified by acetone precipitation and digested with trypsin overnight at 37 °C. Peptides were marked with TMT label reagents according to manufacturer instruction (TMT Mass Tagging kit, Thermo Fisher Scientific), mixed and stored at −80 °C.

### 2.4. Iodoacetyl-Tandem Mass Tag (IodoTMT) Sample Preparation

Albumin depleted serum were acetone-precipitated and resuspended in 1 mL of HENS buffer (100 mM HEPES, 1 mM EDTA, 0.1% Neocuproine and 1% SDS, pH 7.8). One mg of protein was treated and labeled according to the manufacturer’s instructions (Thermo Fisher Scientific). Briefly, cysteine free-thiols were blocked with 20 µL of 1 M MMTS for 30 min at room temperature and proteins were acetone-precipitated overnight to remove MMTS excess. Protein pellets were resuspended in 1 mL HENS buffer. IodoTMT labels were solubilized in 10 µL of methanol, added to each sample together with 20 µL of 1 M sodium ascorbate and samples incubated for 1 h at 37 °C in the dark. Forty µL of 0.5 M dithiothreitol (DTT) was added and samples were incubated for 15 min at 37 °C, in the dark. After mixing and acetone precipitation protein pellets were resuspended in HENS buffer. Then 200 µL of 0.5 M iodoacetamide was added, samples were incubated 1 h at 37 °C in the dark, and acetone-precipitated. Samples were then resuspended in 50 mM AMBIC buffer and digested overnight with trypsin at 37 °C. Digestion was stopped with 250 μL of 10% trifluoroacetic acid (TFA) and peptides were purified with C18 solid-phase extraction columns. After lyophilization and solubilization in 1× TBS, mixtures were enriched by mixing samples with anti-TMT resin (Thermo Fisher Scientific) and incubated at 4 °C overnight. After TBS and water washing, IodoTMT enriched labeled peptides were eluted with TMT elution buffer (Thermo Fisher Scientific). Peptides were lyophilized and stored at −80 °C.

### 2.5. TMT and IodoTMT LC-MS/MS Analyses

All samples were analyzed using a Dionex Ultimate 3000 nano-LC system (Sunnyvale CA, USA) connected to Orbitrap Fusion Tribrid Mass Spectrometer (Thermo Fisher Scientific) equipped with nano electrospray ion source. Peptide mixtures were pre-concentrated onto an Acclaim PepMap 100—100 μm × 2 cm C18 (Thermo Fisher Scientific) and separated on EASY-Spray column ES802A, 25 cm × 75 μm ID packed with Thermo Fisher Scientific Acclaim PepMap RSLC C18, 3 μm, 100 Å using mobile phase A (0.1% formic acid in water) and mobile phase B (0.1% formic acid in acetonitrile) at a flow rate of 0.300 μL/min. MS spectra were collected in positive ion mode, in the data-dependent (DDA)—Synchronous Precursor Selection (SPS)—MS/MS/MS (MS3) mode. Peptides were ionized with a spray voltage of 1600 kV. The instrument method included Orbitrap MS1 scans (resolution of 120,000; mass range 375–1500 *m*/*z*; automatic gain control (AGC) target 4 × 10^5^, max injection time of 50 ms). During the MS2 analyses, precursor ions were filtered according to charge state (required > 1 z), dynamic exclusion (60 s with a ±10 ppm window), and monoisotopic precursor selection. Precursors were isolated with quadrupole mode using a width of 0.7 *m*/*z* and were fragmented by collision-induced dissociation (CID) followed by ion trap MS2 scans (CID collision energy of 35%; AGC target 1 × 10^4^; turbo ion trap scan rate; max injection time of 50 ms). Quantitative SPS-MS3 scans operating in data-dependent mode with precursor selection range 400–1200 *m/z* and 10 SPS precursors were selected; For the MS3 scan, the MS1 precursor was isolated using a 2 *m/z* wide window (resolution of 30,000; HCD collision energy of 65%; scan range 100–500 *m/z*; AGC target 5 × 10^4^; max injection time of 54 ms).

LC-MS/MS data were analyzed by MaxQuant software (version 2.0.3.1, Max Plank Institute of Biochemistry, Munich, Germany), with the following parameters: reporter ion MS3 (label: TMT 6plex or iodoTMT 6plex), trypsin specific (with up to two missed cleavages), human UniProt sequence database (UP000005640, release March 2021). For TMT analysis, Carbamidomethylcysteine was set as a fixed modification, N-terminal acetylation, and methionine oxidation as variable modifications. For iodoTMT analysis, carbamidomethylation at Cys was removed from fixed modifications being contradictory to the label setting. PSM and Protein FDR were set to 1%. Results were investigated with Perseus (version 2.0.3.0, Max Plank Institute of Biochemistry). For each experimental group, only proteins identified in at least 75% of samples were considered. Statistically significant differences were computed by One-way repeated measures ANOVA and Tukey’s test (*p* < 0.05).

### 2.6. Sphingolipids Extraction

Sphingolipids were extracted from sera according to previously published paper [22]. Briefly, 100 μL of serum for each sample was mixed with 1.5 mL of a 0.01% (*w*/*v*) BHT, methanol/chloroform 2:1 solution, fortified with internal standards: sphingomyelin (d18:1/12:0), ceramide (d18:1/12:0) and glucosyl (β)ceramide (d18:1/12:0) (AVANTI polar lipids, Alabaster, AL, US). Mixes were extracted overnight at 48 °C under shaking. After extraction, 0.15 mL of KOH 1 M was added and samples were incubated at 37 °C for two hours. Solutions were neutralized with 0.15 mL of acetic acid 1 M and dried under a nitrogen stream. Sphingolipids were resuspended in methanol, transferred to a clean tube and dried using speedvac. Samples were resuspended in 0.15 mL of methanol and after centrifugation at 10,000× *g* for 3 min, supernatants were stored in glass vial at −80 °C.

### 2.7. Sphingolipids LC-MS/MS Analysis

Ten μL of sphingolipid extracts was injected, separated and analyzed using a Waters Acquity UPLC system coupled to a Waters Synapth G2-Si (Waters, Milford, MA, USA) operating in positive electrospray ionization mode. Full scans were obtained in 50 to 1500 Da windows. Accuracy and reproducibility were maintained by employing an independent reference spray via LockSpray. A C8 Acquity UPLC BEH (Waters) 100 mm × 2.1 mm id, 1.7 µm C8 column was used to separate sphingolipid extracts by a ammonium formate/water and ammonium formate/methanol gradient. Compounds were identified based on mass accuracy with error < 5 ppm, and retention time compared to a standard (±2%), and MS/MS spectra of common fragments. Mass spectra were analyzed by MassLynx™ 4.2 Software (Waters), and lipids were annotated as lipid subclasses as follows (sphingosine backbone/number of carbon atoms of the fatty acid: number of unsaturation of the fatty acid). MS/MS spectra were acquired and assigned as species based on precursor ions and product ions *m/z* 264.268 and *m/z* 266.286, corresponding to sphingosine backbone (d18:1) and dihydrosphingosine backbone (d18:0), respectively.

## 3. Results

### 3.1. Tandem Mass Tag Proteomic Analysis

Tandem mass tag LC-MS/MS analysis provided a dataset of 225 proteins in the placebo and cocktail-treated groups. After data filtration, 158 and 176 proteins from placebo and cocktail groups were detected in at least 75% of subjects, resulting in an 89.77% proteome overlap.

**Figure 1:**
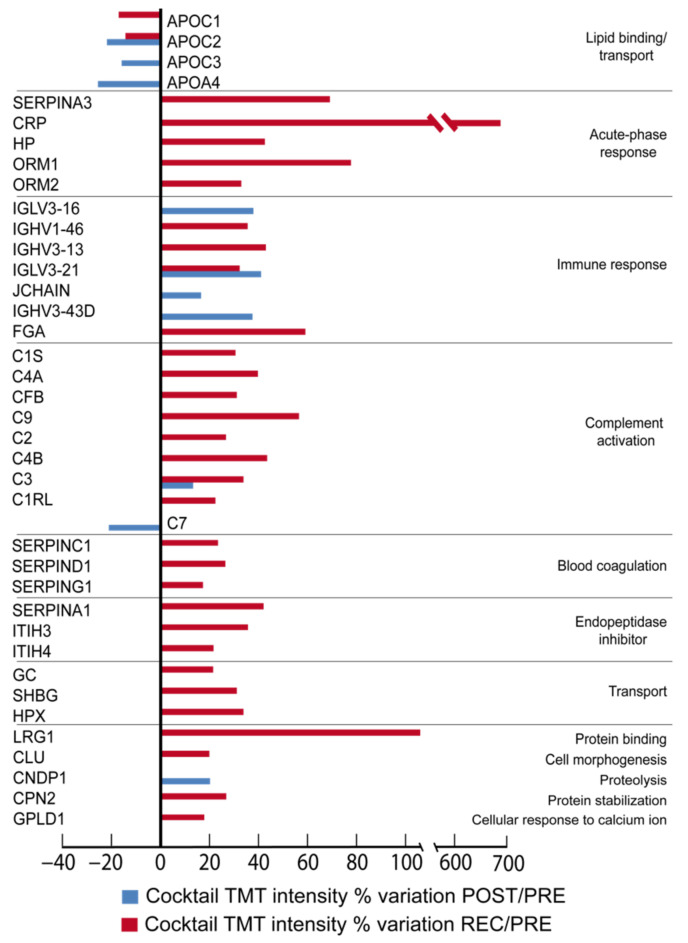
TMT LC-MS/MS proteomic analysis results from sera samples of cocktail-treated BR group. Differential abundant proteins are expressed as histograms representing their intensity as a percentage of variation from PRE. Blue bars represent variation identified in the POST/PRE, while red bars are alterations identified in the REC/PRE comparison. One-way repeated measures ANOVA and Tukey’s test, *n* = 5, *p*-value < 0.05.

**Figure 2:**
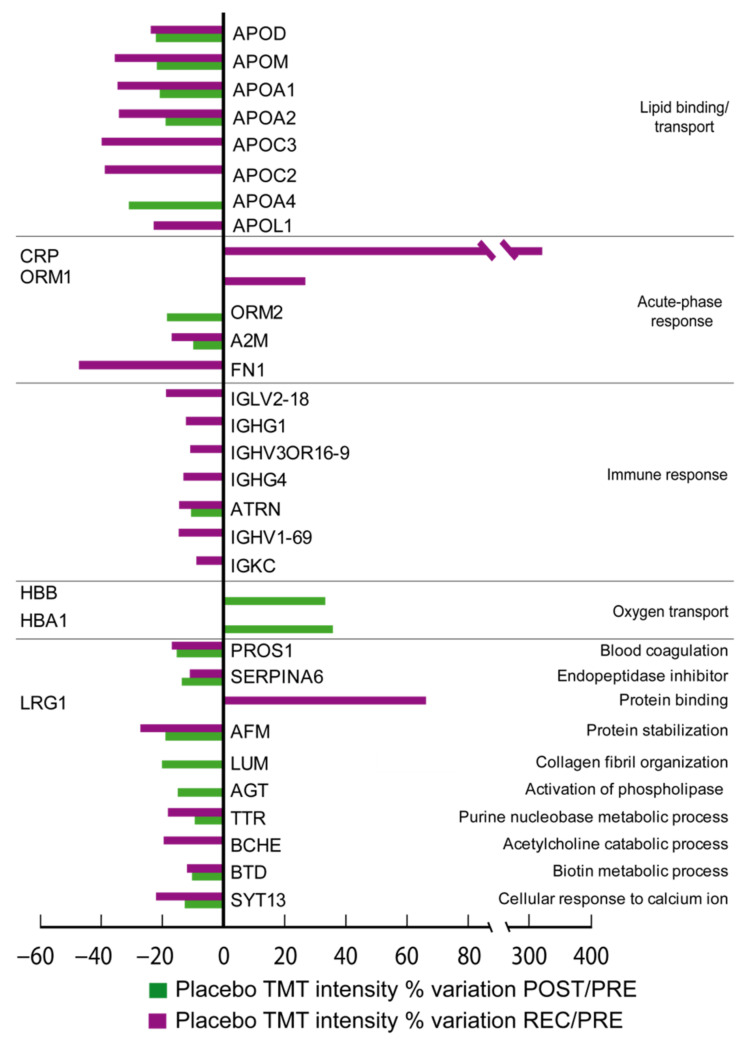
TMT LC-MS/MS proteomic analysis results from sera sample of placebo-treated BR group. Differential abundant proteins are expressed as histograms representing their intensity as a percentage of variation from PRE. Green bars represent variation identified in the POST/PRE, while purple bars represent alterations identified in the REC/PRE comparison. (One-way repeated measures ANOVA and Tukey’s test, *n* = 5, *p*-value < 0.05).

In Figure 1 (Cocktail) and Figure 2 (Placebo), the differentially expressed proteins grouped according to their biological function can be appreciated. By comparing the ratio of differential expression of POST vs. PRE (POST/PRE) and REC vs. PRE (REC/PRE), 39 proteins were identified as changed in the cocktail-treated group, while 32 proteins were significantly changed in the placebo group.

Of the 39 differentially expressed proteins in the cocktail group in POST/PRE and REC/PRE, 5 decreased after BR, while 34 increased. One protein of the complement activation complex, complement component C7 (C7), and three lipid binding/transport proteins, apolipoproteins C-II (APOC2), C-III (APOC3) and A-IV (APOA4), decreased in POST/PRE.

Results from REC/PRE indicated changes in 32 proteins including the lipid binding protein APOC2 and apolipoprotein C-I (APOC1), which decreased. Proteins related to proteolysis Beta-Ala-His dipeptidase (CNDP1), activation of the complement component C3 (C3) and immune response, immunoglobulin heavy variable 3-43D (IGHV3-43D), immunoglobulin J chain (JCHAIN), immunoglobulin lambda variable 3-21 and 3-16 (IGLV3-21, IGLV3-16) increased in the POST/PRE.

Conversely, acute-phase response proteins alpha-1-antichymotrypsin (SERPINA3), C-reactive protein (CRP), haptoglobin (HP), alpha-1-acid glycoprotein 1 (ORM1), alpha-1-acid glycoprotein 2 (ORM2), antithrombin-III (SERPINC1), heparin cofactor 2 (SERPIND1), plasma protease C1 inhibitor (SERPING1) and immune response proteins immunoglobulin heavy variable 1-46 and 3-13 (IGHV1-46, IGHV3-13), IGLV3-21 and fibrinogen alpha chain (FGA) increased in the REC/PRE. The increase in the REC/PRE phase was also found for the following: complement activation proteins complement C1s subcomponent (C1S), complement C4-A (C4A), complement factor B (CFB), complement component C9, C2 and C3 (C9, C2, C3), complement C4-B (C4B), and complement C1r subcomponent-like protein (C1RL); endopeptidase inhibitors alpha-1-antitrypsin (SERPINA1), inter-alpha-trypsin inhibitor heavy chain H3 and H4 (ITIH3, ITIH4); transport proteins vitamin D-binding protein (GC), sex hormone-binding globulin (SHBG) and hemopexin (HPX). The REC/PRE was also characterized by an increase in protein binding leucine-rich alpha-2-glycoprotein (LRG1), cell morphogenesis clusterin (CLU), protein stabilization carboxypeptidase N subunit 2 (CPN2) and cellular response to calcium ion phosphatidylinositol-glycan-specific phospholipase D (GPLD1).

In placebo-treated subjects, 27 out of 32 differentially expressed proteins decreased and 5 increased after BR. Notably, proteins related to lipid binding/transport decreased. Specifically, apolipoproteins D (APOD), M (APOM), A-I (APOA1), A-II(APOA2) and A-IV(APOA4) decreased in the POST/PRE comparison. APOD, APOM, APOA1, APOA2, APOC2, APOC3, and apolipoprotein L1 (APOL1) decreased in the REC/PRE. Immune response proteins decreased during BR in the placebo group, including attractin (ATRN), a protein involved in immune cell clustering during inflammatory response, which decreased in the placebo POST/PRE while immunoglobulin lambda variable 2-18 (IGLV2-18), immunoglobulin heavy constant gamma 1 and 4 (IGHG1, IGHG4), immunoglobulin heavy variable 3OR16-9and 1-69 (IGHV3OR16-9, IGHV1-69), ATRN, and immunoglobulin kappa constant (IGKC) decreased in the placebo REC/PRE.

Acute-phase response proteins ORM2 and alpha-2-macroglobulin (A2M) also decreased in the placebo group in the POST/PRE, and fibronectin (FN1) decreased in the REC/PRE, whereas CRP and ORM1 increased in the recovery phase.

Protein involved in blood coagulation (vitamin K-dependent protein S, PROS1), inhibition of endopeptidases (corticosteroid-binding globulin, SERPINA6), transport of vitamin E in body fluids (afamin, AFM), metabolic processing (transthyretin, TTR and biotinidase, BTD) and cellular response to calcium ions synaptotagmin-13 (SYT13) decreased in POST/PRE and remained decreased in REC/PRE. Lumican (LUM) involved in collagen organization and angiotensinogen (AGT) involved in activation of phospholipases decreased in POST/PRE only. Proteins related to oxygen transport (hemoglobin subunit beta, HBB) and hemoglobin subunit alpha (HBA1) increased in the POST/PRE. LRG1 and acute-phase proteins CRP and ORM1, similarly to cocktail-treated subjects, increased in REC/PRE, while cholinesterase (BCHE) and A2M decreased in the same comparison.

### 3.2. Cocktail-Induced Adaptation in Serum Sphingolipidome

**Figure 3:**
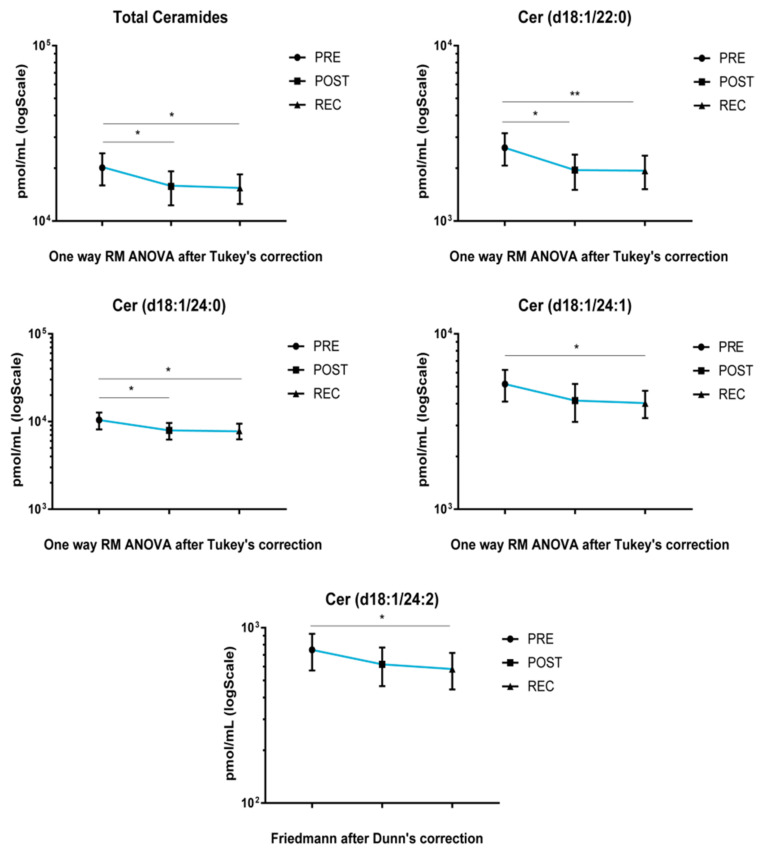
LC-MS analysis results for ceramides in serum of cocktail-treated subjects. Results are expressed as pmol/mL in Log scale (PRE n. 10, POST n. 10, REC n. 10). *p*-values are indicated as: * *p*-value < 0.05 and ** *p*-value < 0.01.

Upon cocktail treatment, total ceramide and Cer acyl chains d18:1/22 and d18:1/24:0 decreased after BR, both in POST vs. PRE and REC vs. PRE. Ceramide acyl chains d18:1/24:1 and d18:1/24:2 decreased in REC vs. PRE comparison (Figure 3).

**Figure 4:**
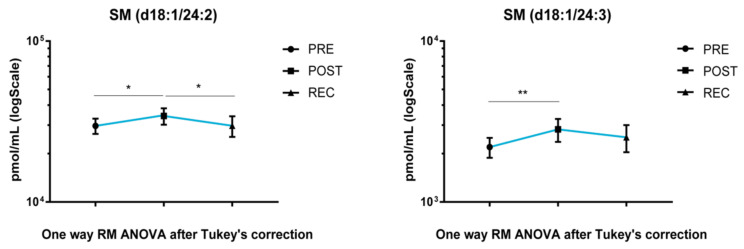
LC-MS analysis results for sphingomyelins in cocktail-treated subjects’ serum. Results are expressed as pmol/mL in Log scale (PRE n. 10, POST n. 10, REC n. 10). *p*-values are indicated as: * *p*-value < 0.05 and ** *p*-value < 0.01.

SM acyl chains d18:1/24:2 and d18:1/24:3 increased after BR in POST vs. PRE upon cocktail treatment. Acyl chain d18:1/24:2 decreased in the REC vs. POST comparison and returned to PRE levels (Figure 4).

### 3.3. BR-Induced Adaptation in Serum Sphingolipidome

**Figure 5:**
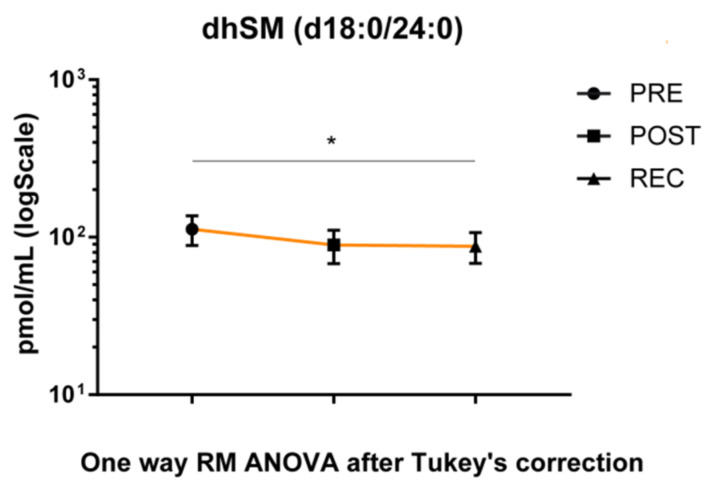
LC-MS analysis results for dhSM (d18:0/24:0) in serum of placebo-treated subjects. Results are expressed as pmol/mL in Log scale (PRE n. 10, POST n. 10, REC n. 10). *p*-value is indicated as: * *p*-value < 0.05.

The dhSM’s acyl chain d18:0/24:0 was the only species with a statistically significant change in the placebo group during BR, decreasing in the REC vs. PRE comparison (Figure 5).

### 3.4. Iodo-Tandem Mass Tag S-Nitrosoproteome Assessment

The iodoTMT protocol, in conjunction with LC-MS/MS analysis, allowed us to identify and quantify 45 *S*-nitrosylated (*S*-NO) proteins.

**Figure 6:**
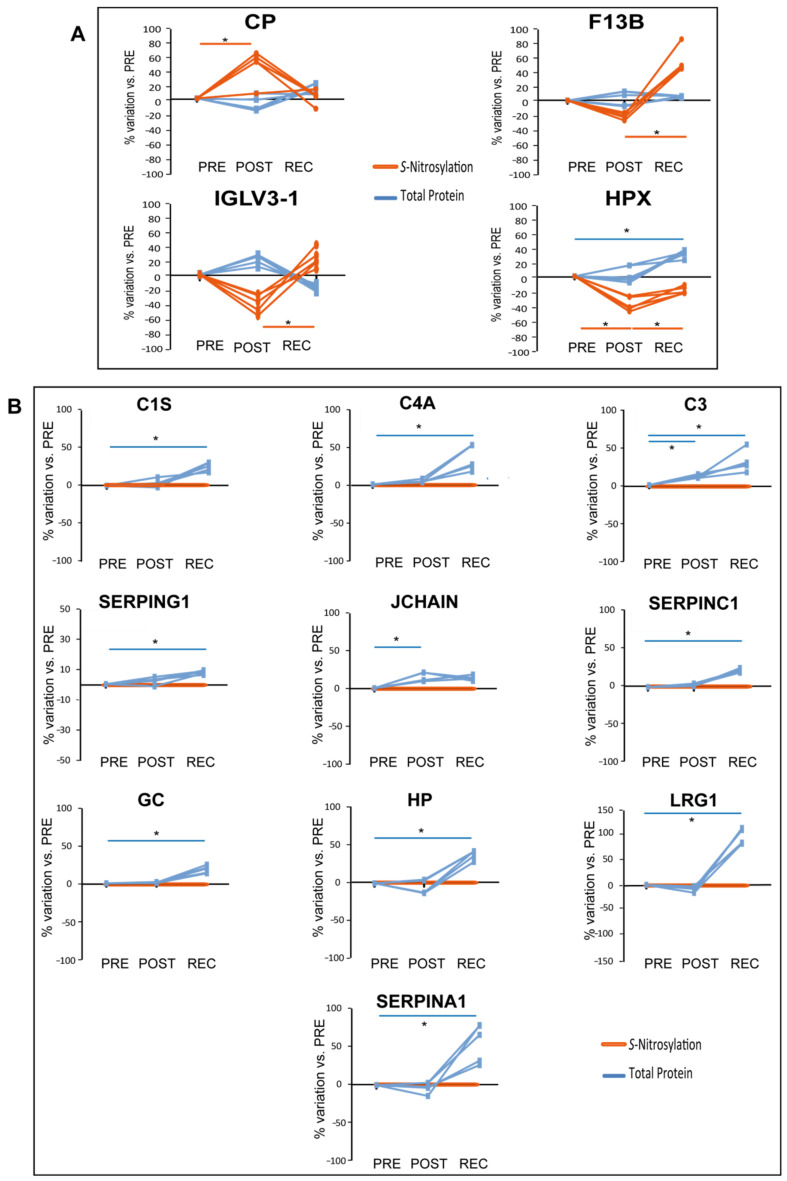
Results from iodoTMT LC-MS/MS proteomic analysis of cocktail-treated subjects. Differentially abundant proteins from TMT and iodoTMT analyses are expressed as a percentage of variation from PRE. Blue lines refer to total protein, while orange lines to protein *S*-Nitrosylation. Significant alterations in protein *S*-nitrosylation (**A**) and significant alteration in total protein levels (**B**). Protein *S*-nitrosylation levels in (**B**) are normalized to 0% for graphical comparison. (One-way repeated measures ANOVA and Tukey’s test, *n* = 5, * *p*-value < 0.05).

When comparing differential levels of S-nitrosylation of proteins identified by iodoTMT analysis in cocktail-treated subjects in POST/PRE and REC/PRE, 4 out of 45 proteins were differentially nitrosylated, as shown in Figure 6A.

In the cocktail-treated group, S-nitrosylation of Ceruloplasmin (CP) increased in POST/PRE while HPX S-nitrosylation decreased in POST/PRE and increased in REC/POST. HPX total protein levels increased in REC/PRE, while levels of nitrosylation decreased, further emphasizing the decrease of *S*-nitrosylation of this protein in REC/PRE. The statistically significant increment of *S*-nitrosylation detected for HPX in REC/POST was also observed for the Coagulation factor XIII B chain (F13B) and for the Immunoglobulin lambda variable 3-1 (IGVL3-1).

Of the 39 differentially abundant proteins identified in the cocktail-treated group, 10 were found to be *S*-nitrosylated in the same group as graphically shown in Figure 6B. In this context, when statistically significant variations occur at the total protein level while non-significant variations occur in the *S*-nitrosylation pattern, they can reveal differences in protein *S*-nitrosylation. Total protein levels of several serum proteins increased in the POST/PRE (C3 and JCHAIN) and REC/PRE (C1S, C4A, C3, SERPING1, SERPINC1, GC, HP, LRG1, and SERPINA1); however, their levels of *S*-nitrosylation did not change, indicating that the ratio of *S*-NO/total protein decreased.

**Figure 7:**
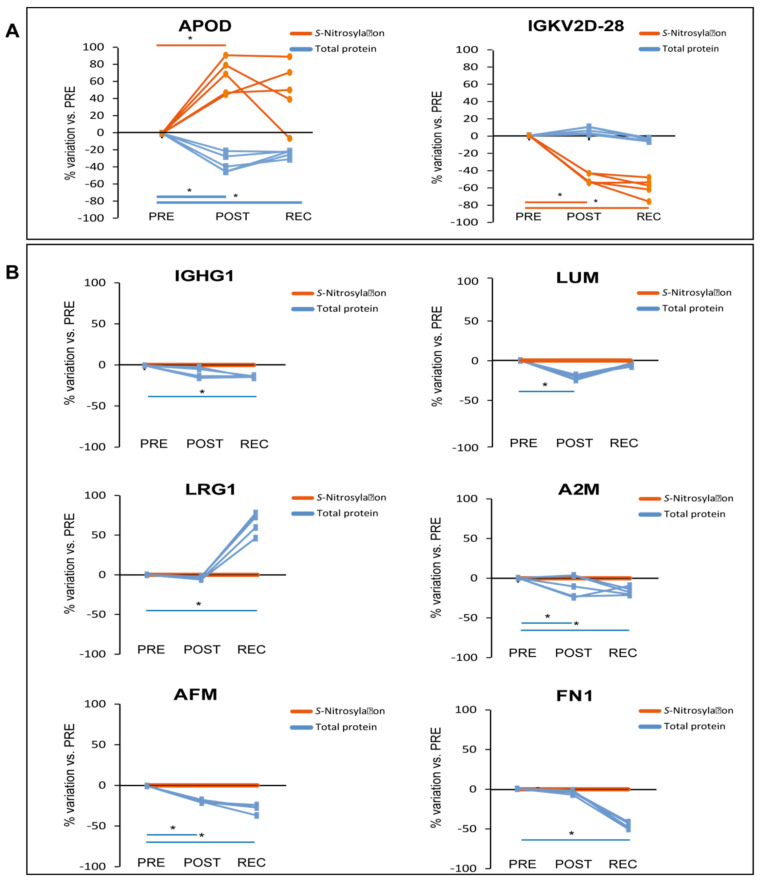
Results from iodoTMT LC-MS/MS proteomic analysis of placebo-treated subjects. Differentially abundant proteins from TMT and iodoTMT analyses are expressed as a percentage of variation from PRE. Blue lines refer to total protein, while orange lines to protein *S*-Nitrosylation. Significant alterations in protein *S*-nitrosylation (**A**) and significant alteration in total protein levels (**B**). Protein *S*-nitrosylation levels in (**B**) are normalized to 0% for graphical comparison. (One-way repeated measures ANOVA and Tukey’s test, *n* = 5, * *p*-value < 0.05).

Two proteins in the placebo group showed a different trend of *S*-nitrosylation after BR as shown in Figure 7A. 

APOD *S*-nitrosylation increased in the POST/PRE while Immunoglobulin kappa variable 2D-28 (IGKV2D-28) nitrosylation decreased in POST/PRE and further decreased in the REC/PRE. Interestingly, the total level of APOD decreased in both POST/PRE and REC/PRE, indicating a further increase of S-NO/APOD ratio (Figure 7A).

Considering total protein and *S*-nitrosylation levels in the placebo group, 7 out of the 32 differentially expressed proteins were *S*-nitrosylated (APOD, IGHG1, LUM, LRG1, A2M, AFM and FN1).

APOD was the only one that decreased its level while its S-nitrosylation increased with statistical significance. In the placebo group (Figure 7B), total protein level of A2M and AFM decreased in REC/PRE and POST/PRE, LUM decreased in POST/PRE whereas IGHG1 and FN1 decreased in REC/PRE. LRG1, similarly to cocktail-treated group, was the only protein increased in REC/PRE, indicating a decreased S-NO/total protein ratio also found in several other serum proteins from the cocktail BR group.

## 4. Discussion

This study provided results from human blood serum proteome, nitrosoproteome and sphingolipids’ imbalance in prolonged BR with and without nutritional intervention by an antioxidative mixed cocktail treatment (Toulouse COCKTAIL study). The general aim was to find putative circulating biomarkers able to monitor the effects of prolonged unloading of the human body. Results indicated that changes induced by daily treatment with a cocktail containing 741 mg of polyphenols, 138 mg of vitamin E, 80 μg of selenium, and 2.1 g of ω-3, affect two main patterns: lipid transport and nitrosative stress.

After 60 days of BR the TMT differential proteomic study of serum proteins indicated changes mainly concentrated on major apolipoproteins, which decreased after BR, leading us to hypothesize an HDL decrease and a possible cholesterol efflux imbalance. Specifically, placebo BR subjects showed decreased levels of proteins associated with HDL: APOA1 (which is the major component of HDL), APOA2 (the second main component of HDL), APOL1, APOD and APOM. In placebo-treated subjects other apolipoproteins also associated with VLDL decreased, namely APOC2, APOC3, and APOA4. Furthermore, placebo-treated subjects showed a decrease in two HDL-associated proteins [23]: ORM2 and TTR. Conversely, the cocktail-treated subjects showed a decrease of apolipoproteins mainly associated with VLDL [24], specifically APOC1, APOC2, APOC3 and APOA4.

The common changes in protein abundance of APOC2, APOC3 and APOA4 observed in the two groups identified a subset of serum apolipoproteins that were affected by BR but not influenced by the antioxidant cocktail treatment. The marked decrease of serum apolipoprotein levels associated with HDL in placebo subjects confirms previous studies performed after short term BR (20, 21 and 35 days, respectively), in which subjects underwent a deterioration of plasma lipid parameters with a decrease in major apolipoprotein APOA1 and APOA2, in HDL_2_, HDL_3_ cholesterol and an increase in VLDL cholesterol and LDL-VLDL triglycerides content [3,6,25]. In cocktail-treated 60-day BR subjects, levels of the main HDL apolipoproteins were not changed. In addition, besides the maintenance of APOA1 and APOA2 levels, other proteins associated with HDL [23] such as C9, C3, C4B, ORM2, SERPINA1, ITIH4 and HPX increased in the recovery phase after cocktail suspension, leading us to hypothesize that the HDL levels themselves are, if not increased, at least maintained in the cocktail-treated BR subjects. The marked difference between cocktail and placebo BR groups suggests that the cocktail exerted a favorable effect in the maintenance of a physiological lipemic profile in BR subjects. However, further investigation will be necessary, particularly regarding the recovery phase after BR in which subjects underwent a sudden cocktail deprivation in conjunction with the remobilization protocol. The cocktail nutritional deprivation post BR, for example, stimulated the acute phase response, the immune response, the complement activation, and generally the response at the whole-body level, indicating that investigation of a recovery phase under cocktail treatment should be compared with recovery with cocktail deprivation, to precisely define the role of this supplement and establish a more refined protocol of cocktail treatment. What is of note is that the lipemic profile post BR was not influenced. The decline in HDL cholesterol and increase in VLDL triglyceride levels are known risk factors for CVD and are correlated with physical inactivity including BR [3]. It is generally accepted that HDLs are cardio-protective, since they promote the removal of cholesterol excess from plaque macrophage foam cells [26]. Prolonged BR reduces plasma HDL levels and markedly suppresses HDL cholesterol efflux capacity, thus hampering the removal of cholesterol from macrophages [27]. It has been described that at the systemic level, the ω-3 cocktail used in the present study fully prevented hypertriglyceridemia, the drop in fasting HDL, and total fat oxidation following a 20-day study of step reduction [19]. This assumption is now also confirmed by our present results from 60 days of BR.

Another important point is that countermeasures commonly adopted to reduce muscle loss during BR, such as resistive vibration exercises, did not correct for HDL levels [6]. Thus, the cocktail treatment sustaining HDL levels and protecting subjects from CVD allows us to hypothesize that combined protocols of resistive countermeasures and cocktail administration will be advisable in extended periods of disuse such as BR and possibly also in long space flight missions.

Our previous proteomic study on muscle sample extracts of the same Cocktail study subjects identified prevention of nitrosative stress and muscle deterioration in cocktail-treated subjects, highlighting a reduction of inflammatory pathways and development of skeletal muscle/muscle contraction [20]. Specifically, muscle proteins related to inflammatory pathways (HP, C4A, C3 and IGHG3) decreased, while proteins associated with the development of skeletal muscle/muscle contraction prelaminin A/C (LMNA), neuroblast differentiation-associated protein (AHNAK), tropomyosin alpha-3 chain (TPM3), myosin-1(MYH1), myosin light chain 1/3 (MYL1), myosin-7 (MYH7), myosin-binding protein C (MYBPC1), ryanodine receptor 1 (RYR1) and sarcoplasmic reticulum histidine-rich calcium-binding protein (HRC) increased upon cocktail treatment [20]. In serum, increased levels of HP, C4A and C3 can suggest their cellular release into the blood stream and can be potential biomarkers to monitor the cocktail treatment.

The scope of the present study is not only to describe changes at serum level induced by immobilization in healthy male subjects but also to find molecules easily detectable in the clinical laboratory. Sphingolipids are a structural component of cell membranes and behave as signaling molecules in a number of physiological and pathological processes [22,28,29,30]. Furthermore, they are easily quantifiable by mass-spectrometry, with low costs, and their detection can be easily implemented in clinical settings. It is generally accepted that sphingolipids play a peculiar role in CV dysfunction [31,32]. A recent study indicated an unchanged ceramide profile in young adults after short-duration BR (5 days) [33]. Sphingolipids, both ceramides and sphingomyelins, being scarcely soluble in plasma, are mainly associated with plasma lipoproteins or carried out by micro-vesicles. In particular, ceramide bound to apolipoproteins appears to be mainly present in VLDL and LDL (about 85% of the total) [34], while sphingomyelins are mainly associated with LDL (50%) and HDL (40%) [34]. Results from the serum sphingolipid profile indicated no specific changes in placebo BR subjects, except for a decrease of dhSM d18:0/24:0 chain in REC/PRE comparison, confirming results from a previous study in young subjects [33]. Conversely, cocktail-treated BR subjects showed a decrease of total ceramide levels and of C22, C24, C24:1 and C24:2 Cer’s acyl chains, which were also decreased in the recovery phase. By contrast, levels of sphingomyelin C24:2 and 24:3 in the same subjects increased during BR and recovered to basal level in the REC/PRE phase. As highlighted by Ferchaud-Roucher et al. [15], ω-3 supplement decreased serum VLDL and APOB100 content while improving their sphingolipid content, supporting the role of ω-3 in the maintenance of lipemic homeostasis.

From our present results, we can postulate that cocktail nutritional countermeasure impacts on both serum apolipoproteins and sphingolipids following BR, being the latter unchanged in post BR placebo subjects. Further studies should be performed to understand the reasons behind this decrement during treatment and its significance in otherwise healthy subjects.

The other aspect addressed by our study is the protection exerted by the cocktail towards nitrosative stress elicited by reactive oxidative and nitrosative species. Systemic or tissue specific nitric oxide (NO) imbalance can promote inflammation, oxidative stress, endothelial dysfunction, and tissue injury. Moreover, RONS are potential contributors to both chronic kidney disease (CKD) and CVD progression [35,36,37]. In the placebo BR group, the ratio of *S*-NO/total proteins increased for several proteins (IGHG1, A2M, AFM, FN1 and LUM), suggesting that these proteins are over-nitrosylated during BR. At the systemic level we found that in the placebo conditions in BR, despite its reduced protein abundance, APOD is over-nitrosylated.

Collectively, these results indicated that nitrosative stress is present at the systemic level (increased *S*-NO/total protein in blood serum), suggesting that imbalance of serum-related RONS after prolonged BR may be a critical aspect. In vitro and in vivo studies have shown that enzymatic oxidation, lipolysis, and proteolysis can modify HDL and affect the HDL capacity to promote cellular cholesterol efflux [38,39]. The biological significance of the role of APOD over nitrosylation, for example, can be ascribed in this context. In plasma, APOD forms disulfide-linked homodimers and heterodimers with APOA2 [40] and this formation can be impaired by nitrosylation of APOD. Further investigation will also be required to precisely define the role of APOD in cholesterol efflux. In fact, it has been described that in individuals with CVD, the oxidation of HDL results in selective inhibition of cholesterol efflux from macrophages [41]. Cocktail-treated BR subjects appeared to be somehow protected from nitrosative stress as indicated at least by a decreased ratio of *S*-NO/total proteins observed for C1S, C4A, C3, SERPING1, SERPINC1, GC, HP, LRG1, SERPINA1 and JCHAIN. At variance, CP and F13B increased their nitrosylation despite the protein level that turned out to be unchanged. This behavior suggests specific signaling induced by nitrosylation that needs to be further investigated. Ceruloplasmin (CP) is an acute-phase reactant protein involved in iron and copper metabolism [42], with antioxidant and pro-oxidant activity, and regulates NO homeostasis being involved in CP-mediated lipid peroxidation and protein nitration [43,44,45]. It has also been described that in vitro CP is characterized by a NO oxidase activity, controlling the catalytic consumption of NO. When CP plasma levels decrease, diminished plasma NO oxidase activity contributes to keep under control NO oxidation [43,46]. Ceruloplasmin-mediated NO oxidase activity occurs in the three type 1 copper-binding domains of the protein, where NO is oxidated to NO^+^ and Cu^2+^ is reduced to Cu^1+^ [43]. These domains are cysteine-rich [47,48], and we may hypothesize that the elevated CP *S*-nitrosylation observed in our study can interfere with the CP-mediated NO oxidase activity. Further studies will be required at the cellular level to mechanistically address this issue. Conversely, HPX decreased its nitrosylation level despite protein increment that requires further elucidation in future studies, while IGLV3-1 increased its nitrosylation. From the literature, no studies have been performed in immunoglobulin lambda variable nitrosylation and at this point no consideration can be made for this protein. Concerning HPX, it works as a scavenger and transporter of toxic plasma heme and can play a key role in NO homeostasis facilitating NO/O_2_ and NO/peroxynitrite scavenging [49]. HPX sequesters heme, preventing heme-mediated activation of oxidants that induce low-density lipoprotein oxidation [50,51]. Moreover, oxidants that are otherwise activated by hemeproteins become inactive in the presence of HPX, suggesting that these changes can have relevant physiological implications in redox balance homeostasis [52,53].

Collectively these results indicate that the decrease of LDL-associated proteins, unchanged levels of HDL-associated proteins, and the decrease of Cers chains can be hallmarks of the cocktail treatment during long-term BR. Furthermore, specific serum proteins either over- or under-nitrosylated can contribute to redox homeostasis, suggesting that the cocktail treatment exerts a positive effect in keeping redox homeostasis at least under control. On the other hand, the over-nitrosylation of APOD in placebo BR subjects can exacerbate lipid imbalance, further corroborating the positive role of antioxidative cocktail supplementation in preventing oxidative stress following prolonged BR.

This is the first “omic” study addressing the human blood serum proteome, nitrosoproteome and sphingolipidome to evaluate the imbalance induced by BR and the impact of a cocktail treatment to prevent possible immobilization-induced health risks of inactivity and disuse of the human body. However, the study has several limitations that include the small sample size that also characterizes previous BR studies due to their high costs and complexity, and the absence of HDL, LDL and cholesterol assessments not included in the protocol and not achievable due to the low available amount of sera samples for our analyses. Since biological effects regarding many of the serum protein changes and the nutritional outcomes of antioxidative nutritional supplementation following prolonged BR have not been clearly demonstrated, speculations about mechanisms were provided to support our hypothesis based on relevant studies available in the literature. Further experiments will be required to confirm our hypothesis on the role of APOD, CP and HPX in the context of unloading and disuse conditions. However, this study utilizing liquid biopsies can open new avenues not only for bedridden patients but also for astronauts involved in long-duration missions, in which the monitoring of specific proteins (APOA1, APOA2, C9, C3, C4B, ORM2, SERPINA1, ITIH4 and HPX, nitrosylation of APOD, CP, HPX) and Cers chains during the mission could help to better assess potential health risks such as CVD complications.

## Data Availability

Study data from this human study other than those published in this work are under privacy regulations but can be obtained on a case-to case basis upon reasonable request from the corresponding author.
